# IL-17 Production from T Helper 17, Mucosal-Associated Invariant T, and γδ Cells in Tuberculosis Infection and Disease

**DOI:** 10.3389/fimmu.2017.01252

**Published:** 2017-10-11

**Authors:** Felicity Coulter, Amy Parrish, Declan Manning, Beate Kampmann, Joseph Mendy, Mathieu Garand, David M. Lewinsohn, Eleanor M. Riley, Jayne S. Sutherland

**Affiliations:** ^1^Vaccines and Immunity Theme, Medical Research Council Unit, Banjul, Gambia; ^2^Department of Immunology and Infection, London School of Hygiene and Tropical Medicine, London, United Kingdom; ^3^The University of Manchester, Manchester, United Kingdom; ^4^Pulmonary and Critical Care Medicine, Portland VA Medical Center and Oregon Health & Science University, Portland, OR, United States

**Keywords:** T helper 17, mucosal-associated invariant T, gamma delta, tuberculosis, IL-17 superfamily

## Abstract

IL-17-producing cells have been shown to be important in the early stages of *Mycobacterium tuberculosis* (Mtb) infection in animal models. However, there are very little data on the role of IL-17 in human studies of tuberculosis (TB). We recruited TB patients and their highly exposed contacts who were further categorized based on results from an IFN-γ-release assay (IGRA): (1) IGRA positive (IGRA^+^) at recruitment (latently TB infected), (2) IGRA negative (IGRA^−^) at recruitment and 6 months [non-converters (NC)], and (3) IGRA^−^ at recruitment and IGRA^+^ at 6 months (converters). Whole blood was stimulated with mycobacterial antigens and analyzed using T helper (Th) 17 multiplex cytokine assays. Th17, Vγ9Vδ2^+^, and CD161^++^Vα7.2^+^ mucosal-associated invariant T (MAIT) cells were analyzed by flow cytometry. The majority of IL-17 was produced by CD26^+^CD4^+^ Th17 cells (median 71%) followed by γδ T cells (6.4%) and MAIT cells (5.8%). TB patients had a significantly lower proportion of Th17 cells and CD4^+^CD161^+^Vα7.2^+^ cells producing both IL-17 and IFN-γ compared to LTBI subjects. IGRA NC had significantly lower levels of CD26^−^CD4^+^ and CD8^+^ MAIT cells producing IL-17 compared to IGRA C but had significantly higher levels of IL-17A, IL-17F, IL-21, and IL-23 in ESAT-6/CFP-10-stimulated supernatants compared to IGRA C. These data provide new insights into the role of IL-17 and IL-17-producing cells at three key stages of the Mtb infection spectrum.

## Key Points

- Tuberculosis patients have fewer circulating CD4^+^CD161^+^Vα7.2^+^ cells producing both IFN-γ and IL-17 compared to latently infected individuals.
- Highly exposed contacts who remain uninfected had lower levels of circulating CD8^+^ mucosal-associated invariant T cell-producing IL-17 compared to those who become infected.

## Introduction

Despite recent advances in tuberculosis (TB) diagnosis and treatment, it still remains a major infectious disease killer in resource-poor settings. In 2015, 10.4 million new cases were diagnosed resulting in 1.8 million deaths and over 50 million new infections (1). A major gap in knowledge for development of new vaccines is an understanding of what constitutes natural protective immunity. While there have been several insights through animal models, translation of these observations to humans is severely lacking, hampering the development of protective vaccines.

Interestingly, of the estimated 2–3 billion people infected with the causative pathogen, *Mycobacterium tuberculosis* (Mtb), only 10% will develop active disease during their lifetime, while the remaining 90% are considered to be latently TB infected (LTBI). Thus, comparison of latently infected individuals with active TB cases may give some insight into factors that protect from development of disease, while analysis of individuals that remain tuberculin skin test (TST) or interferon-gamma release assay (IGRA) negative despite high exposure to TB may give some insight into factors that protect against initial infection. Studies have indicated a protective role for T helper (Th) 17 (2), mucosal-associated invariant T (MAIT) (3), and γδ T cells (4) in TB, but this appears to depend on the stage of Mtb infection analyzed and whether mice or humans are studied.

Th17 cells have been implicated in the pathology of TB by inducing neutrophilic inflammation and mediating tissue damage (5). However, Th17 cells are capable of mediating both antimicrobial and pro-inflammatory responses, suggesting that their role during primary TB infection may be complex (5). Interestingly, a study analyzing TST^+^ vs TST^−^ subjects showed a downregulation of IL-17, IL-23, and RORγt (a key transcription factor for Th17 cells) in TST^+^ individuals but no difference in Th1 and Th2 cytokines, suggesting that a paucity of Th17 cells either predisposes to or is a consequence of Mtb infection (6). In addition, circulating levels of IL-17 and IFN-γ have been shown to be lower in patients with active TB than in those with LTBI (7), again suggesting that the lack of IL-17 may either predispose to active TB disease or be a consequence of it. However, other studies have shown that dual production of IL-17 and IFN-γ from multifunctional Th17 cells correlates with disease severity (8). Thus, the role of Th17 cells during primary Mtb infection remains unclear.

Mucosal-associated invariant T cells are the most abundant innate-like T cell in the human body, forming up to 5% of the human peripheral T cell population (9). MAIT cell depletion has recently been shown to increase the likelihood of developing severe bacterial infections (10). MAIT cells have a semi-invariant T cell receptor (TCR) alpha chain (Vα7.2), are restricted by the MHC-related protein 1, and are activated by cells infected with bacteria or yeast (3) and viruses (11). They express high levels of CD161 (c-type lectin) and have been shown to be reduced in the blood of patients and appear in the lung during active TB disease (3, 12).

γδ T cells comprise 1–5% of peripheral blood lymphocytes (13, 14) and occur in a pre-activated differentiation state at high clonal frequencies, allowing for much faster responses compared to other cell types (15). The invariant γδ TCR recognizes phospho-antigens originating from both the host and bacteria (16), and evidence of their contribution to the immune response against TB has grown steadily over the years (17). γδ T cells are well documented as an early source of IL-17 and IFN-γ following a range of immune challenges (18, 19). In healthy adults, the major peripheral blood γδ T cell subset expresses the Vγ9Vδ2 TCR and displays pleiotropic features with IL-17(+) Vγ9Vδ2 T lymphocytes playing a role in inflammation during bacterial meningitis (20).

In this study, we investigated the Mtb antigen-specific production of IL-17 family cytokines in TB patients and their exposed household contacts in Gambia and evaluated the cellular source of IL-17 using multiparameter flow cytometry.

## Materials and Methods

### Participant Information

Adults with smear positive (and culture-confirmed TB) were recruited following written informed consent and followed up to completion of treatment (standard regimen). Their TB-exposed household contacts were also recruited and analyzed for infection status using an in-house IGRA at baseline and 6 months from baseline. They were subsequently classified as IGRA converters (C) or non-converters (NC) (matched for exposure based on sleeping proximity to the index case and smear grade of the index case). Subjects who were IGRA^+^ at recruitment were defined as latently infected (LTBI). Venous blood samples were collected into heparinised vacutainers (Becton Dickinson, USA) and used for whole blood stimulation (for IGRA NC and C only) or separated into PBMC for flow cytometry analysis (all subjects). Only HIV-negative subjects were included in our analysis.

### Sample Collection and Processing

#### Whole Blood Antigen Stimulation

450 µl whole blood was incubated with 50 µl of phosphate-buffered saline (PBS) as a negative control (NIL) or with 50 µl of phytohemagglutinin (PHA; 5 µg/ml) as a positive control, purified protein derivative (PPD), final concentration 10 µg/ml (Staten Serum Institute, Denmark), or early secretory antigenic target 6, culture filtrate protein 10 fusion protein (ESAT-6/CFP-10; EC; final concentration 10 µg/ml; kindly provided by Prof. T. Ottenhoff, Leiden University Medical Center, The Netherlands). Following overnight incubation at 37°C, 5% CO_2_, supernatant was collected and stored at −20°C prior to use.

#### Multiplex Cytokine Arrays

Multiplex immunoassays were carried out using the 15-plex Bio-Plex Pro™ Human Th17 Cytokine Panel (Bio-Rad, CA, USA) according to the manufacturer’s instructions. Analytes measured were IL-1β, IL-4, IL-6, IL-10, IL-17A, IL-17F, IL-21, IL-22, IL-23, IL-25, IL-31, IL-33, IFN-γ, sCD40L, and TNF-α. Briefly, lyophilized standards were reconstituted, and serial dilutions were performed. Coupled beads were diluted in assay buffer, and 50 µl was added to each well of the assay plate. Fifty microliters of diluted standards, blanks, samples (stimulated whole blood supernatants), and controls were added per well. Plates were then incubated at room temperature (RT), with shaking at 350 rpm, for 30 min followed by three washes in wash buffer. Detection antibodies were diluted to 1 in 20 of their original concentration in detection antibody diluent and 25 µl added to each well followed by another 30-min incubation. Following three washes, streptavidin-phycoerythrin (PE) was diluted to 1 in 100 in assay buffer and 50 µl was added to each well. Plates were then incubated for 10 min and washed three times. 125 µl assay buffer was then added to each well and plates were briefly shaken and subsequently read using Magpix plate reader, with Bio-Plex Manager Software (version 6.1; Bio-Rad, Belgium). No significant differences were observed in background levels within or between groups. Thus, all cytokine responses for NIL-stimulated samples were subtracted from those for blood incubated with EC and PPD antigens.

#### Multiparameter Flow Cytometry

Cryopreserved PBMC samples were thawed and resuspended in RPMI + 10% FCS + 0.02% benzonase. After 6 h of rest, cells were counted and resuspended at 0.5–1.0 × 10^6^ cells per test in 500 µl in polystyrene tubes and incubated at 37°C, 5% CO_2_ for 16–20 h with 1× cell stimulation cocktail plus protein transport inhibitors (CSC; eBioscience, USA), containing phorbol 12-myristate 13-acetate (PMA), ionomycin, brefeldin A, and monensin. Negative controls were incubated in the same conditions without the PMA but with a corresponding 1× protein transport inhibitor cocktail (PTI; eBioscience, UK). After overnight stimulation, tubes were centrifuged at 1,500 rpm for 5 min, and the supernatant was removed. Cells were incubated with Live/Dead Aqua (eBioscience, UK) for 10 min at RT in the dark. Cells were washed with 1 ml FACS buffer (PBS, 1% FCS, 0.2% Na Azide, 0.1% EDTA) and centrifuged at 1,500 rpm for 5 min. Supernatant was removed, and a cell surface cocktail of anti-human CD3 allophycocyanin (APC)-cyanine 7, CD8 Alexa Fluor 700, CD161 PE, Vα7.2 APC (all from eBioscience, UK), and CD26- or Vγ9δ2 TCR fluorescein isothiocyanate (kindly provided by Prof Dietrich Kabelitz, Kiel University, Germany) was added and incubated for 15 min at 4°C. Cells were then washed in 1 ml FACS buffer and centrifuged at 1,500 rpm for 5 min; 150 µl of Cytofix/Cytoperm solution was added (Becton Dickinson, USA) and incubated for 15 min at 4°C. Another wash step was performed, and cells were incubated with 1× Perm/Wash buffer (BD, USA) for 20 min at RT in the dark. Cells were washed and centrifuged at 1,800 rpm for 5 min, supernatant removed and intracellular cytokine staining performed with IFN-γ PE-CF594 and IL-17 PE-cyanine 7 made up in 1× Perm/Wash buffer (all from BD, USA). Cells were incubated for 30 min at RT, in the dark, washed once, and resuspended in 300 µl FACS buffer. 200,000 lymphocytes were acquired per sample using a LSR III Fortessa flow cytometer (BD Biosciences, USA) and BD FACSDiva software. Resultant FACS plots were analyzed using FlowJo (Version 10.1; Treestar, USA).

### Data Analysis

Data were analyzed using Prism v7 (GraphPad, CA, USA). Kruskal–Wallis test with Dunn’s post-test comparison (adjusted for multiple testing) was used to compare groups. Wilcoxon matched rank test was used to analyze within each group. Adjustment for age and sex was performed using logistic regression for data comparing TB with LTBI. However, no differences in results were observed with adjustment; thus all results are presented as unadjusted Mann–Whitney *U*-test values.

### Ethics Statement

This study was carried out in accordance with the recommendations of the MRCG/Gambian government joint ethics committee with written informed consent from all subjects. All subjects gave written informed consent in accordance with the Declaration of Helsinki. The protocol was approved by the MRCG/Gambian government joint ethics committee.

## Results

### Characteristics of Study Participants

A total of 41 subjects were included in this study: 11 IGRA NC, 10 IGRA C, 10 LTBI, and 10 active TB (Table 1). The IGRA NC, IGRA C, and active TB were analyzed both at recruitment and 6 months later. The LTBI were analyzed just at recruitment. The median ages for each group were comparable except the LTBI who were significantly younger than the other groups (*p* = 0.0126). In addition, while the majority of subjects were females in the IGRA NC, IGRA C, and LTBI groups; 90% of TB cases were males (*p* < 0.0001).

**Table 1:** Participant information.

	IGRA NC	IGRA C	LTBI	Active TB
*N*	11	10	10	10
Age, median (IQR)	27 (21–34)	29 (24–40)	21 (19–25)	29 (22–46)
Males, *n* (%)	3 (27)	1 (9)	2 (20)	9 (90)

*IGRA, IFN-γ release assay; NC, non-converters; C, converters; TB, tuberculosis; LTBI, latent TB infection; IQR, interquartile range*.

### CD26^+^CD4^+^ Th17 Cells Are the Major IL-17-Producing Cells

**Figure 1:**
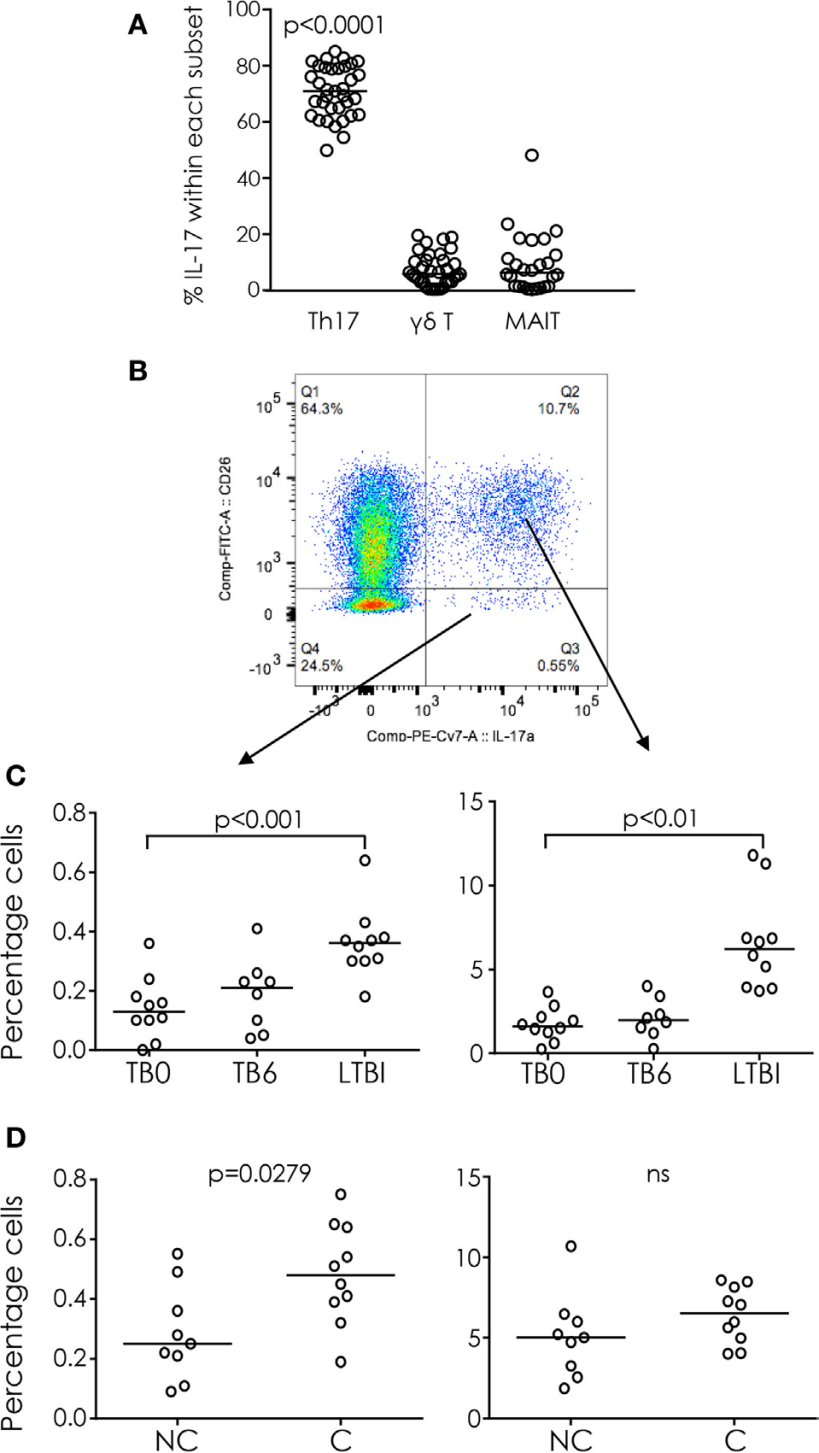
IL-17 producing cells in tuberculosis (TB) infection and disease. **(A)** Percentage of T helper 17 (Th17) cells, γδ T cells, and mucosal-associated invariant T (MAIT) cells within total IL-17^+^ cells (gated from lymphocytes). The majority of IL-17 was produced by Th17 cells. **(B)** Representative FACS plot of IL-17 production from CD26^+^ (Th17; upper right quadrant) and CD26^−^ (lower right quadrant) CD4^+^ cells. **(C)** IL-17 production from CD4^+^CD26^−^ (left) and CD4^+^CD26^+^ (right) cells from TB cases before treatment (TB0) and after treatment (TB6) and latently TB infected (TST^+^). **(D)** IL-17 production from CD4^+^CD26^−^ (left) and CD4^+^CD26^+^ (right) cells from IGRA non-converters (NC) and converters (C). Data were analyzed using Kruskal–Wallis test with Dunn’s post-test comparison **(A,C)** or Mann–Whitney *U*-test **(D)**. Bar indicates median.

CD26 has been shown to be a marker of Th17 cells (21). In all subject groups, the main source of IL-17 after PMA/ionomycin stimulation of purified PBMCs were CD26^+^ Th17 cells (*p* < 0.0001 compared to all other subjects; Figures 1A,B), which accounted for 71% (63–80%) of all IL-17^+^ lymphocytes. γδ T cells and MAIT cells accounted for 6.4% (1.3–14%) and 5.8% (3.0–10.7%) of IL-17^+^ cells, respectively (Figure 1A).

The proportion of Th17 cells was significantly higher among latently infected (LTBI) subjects than among those with active TB before (TB0) or after (TB6) treatment (*p* < 0.001 and *p* < 0.01, respectively; Figure 1C). Interestingly there was no significant difference in the proportion of Th17 cells between IGRA C and IGRA NC, but the proportion of CD4^+^CD26^−^ IL-17^+^ cells was significantly higher in IGRA C compared to NC at baseline (*p* = 0.0279; Figure 1D).

### IL-17 and IFN-γ Dual-Producing Th Cells

**Figure 2:**
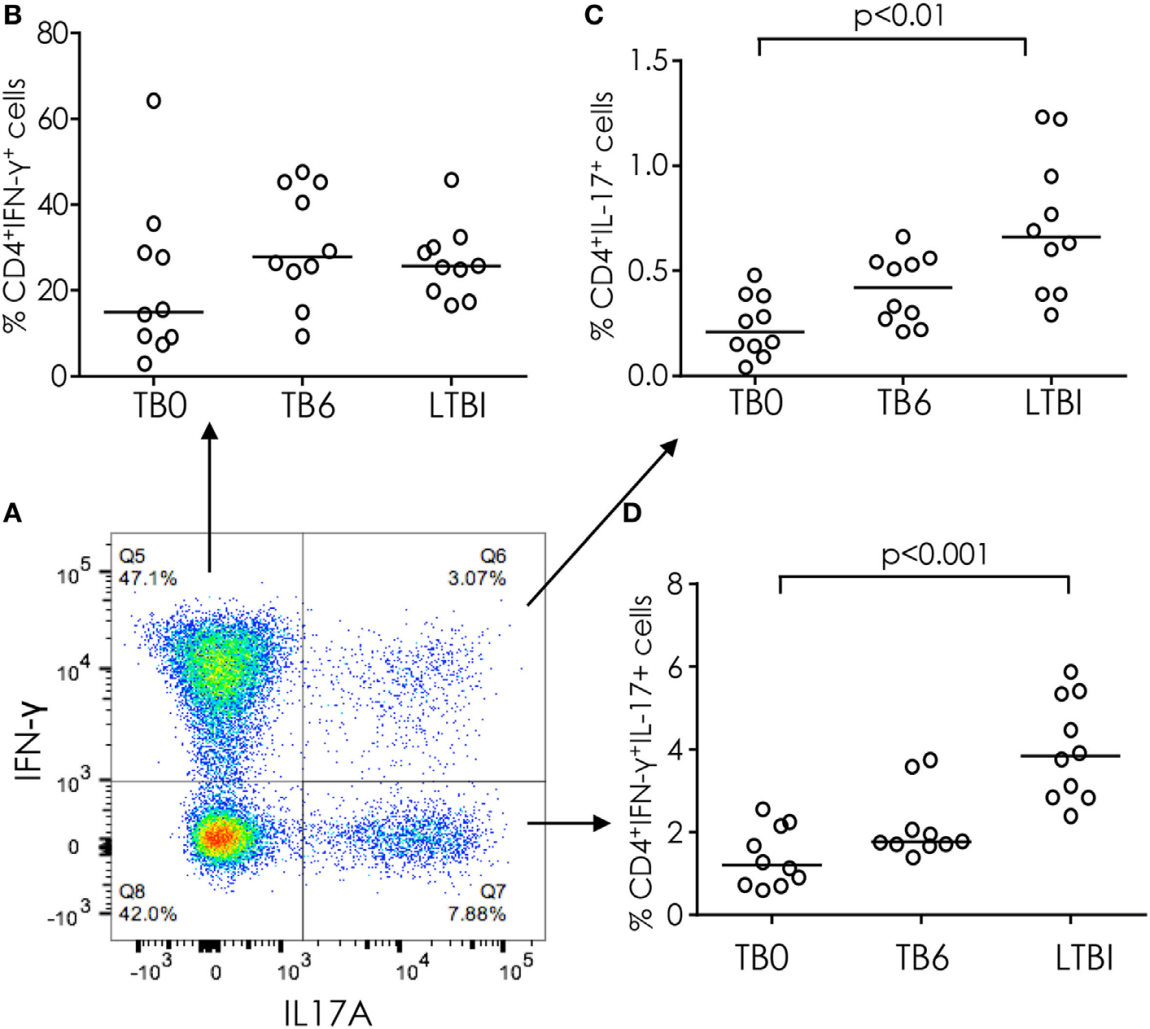
IFN-γ and IL-17 production from CD4^+^ T cells in tuberculosis (TB) infection and disease. **(A)** Representative FACS plot of IFN-γ and IL-17 production from CD4^+^ T cells. **(B)** % CD4^+^ cells producing IFN-γ alone in TB cases before and after treatment and in latently TB infected (from FACS plot top left quadrant). **(C)** % CD4^+^ cells producing both IFN-γ and IL-17 (from FACS plot top right quadrant). **(D)** % CD4^+^ cells producing IL-17 only (from FACS plot bottom right quadrant). Data were analyzed using Kruskal–Wallis test with Dunn’s post-test comparison. Bar indicates median.

CD4^+^ T cells that co-express IL-17 and IFN-γ have been reported to be associated with TB disease severity (8) and are increased in certain autoimmune diseases (22). Although CD4^+^ T cells tended to produce only IFN-γ [median interquartile range (IQR) 37% (27–45%)] or only IL-17 [median (IQR) 4% (2–5%)] a proportion of cells co-produced both cytokines [median (IQR) 1.2 (0.6–1.6%)] (Figure 2A). The proportion of cells producing only IFN-γ did not differ significantly between LTBI and active TB cases (LTBI vs TB0 or TB6) and was not affected by 6 months of TB treatment (TB0 vs TB6) (Figure 2B). However, the proportions of cells producing IL-17 alone or IL-17 in combination with IFN-γ were significantly higher in subjects with LTBI than in those with active TB (*p* < 0.01 and *p* < 0.001, respectively; Figures 2C,D). Although there was an increase in the median percentages of IL-17^+^ and IL-17^+^IFN-γ^+^CD4^+^ T cells after treatment of active TB (TB6 vs TB0) these differences were not statistically significant (Figures 2C,D). No differences in any subset were seen between IGRA NC and C (data not shown).

### CD3^lo^ but Not CD3^bright^ γδ T Cells Produce IL-17

**Figure 3:**
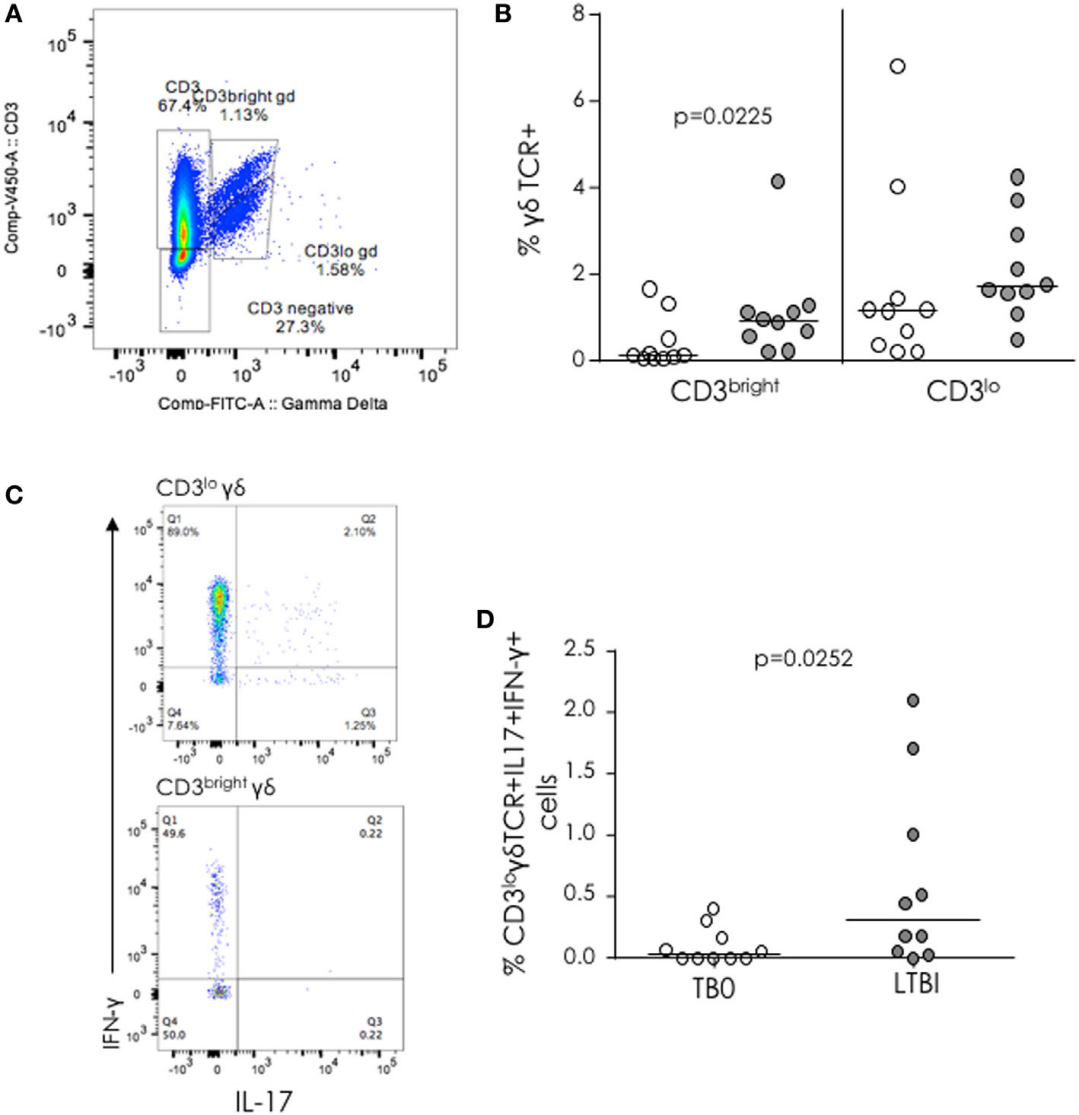
γδ T cells in tuberculosis (TB) infection and disease. **(A)** Representative FACS plot showing Vγ9δ2 TCR^+^ cells with low (lo) and bright CD3 expression. **(B)** Proportion of CD3^bright^ and CD3^lo^ γδ T cells from TB patients (white dots) and latently TB infected (LTBI) (gray dots). **(C)** Representative FACS plots of IFN-γ and IL-17 expression from CD3^lo^ cells. **(D)** % IL-17^+^IFN-γ^+^γδ T cells from TB cases (TB0) and LTBI. Data were analyzed with Mann–Whitney *U*-test. Bar indicates median.

IL-17(+) Vγ9Vδ2 T lymphocytes have been shown to contribute to inflammation during bacterial infections (23) and to respond preferentially to Mtb antigens (24). Therefore, we analyzed Vγ9δ2 T cells in TB cases and LTBI contacts and saw two distinct populations: CD3^bright^ and CD3^lo^ (Figure 3A). These CD3^lo^ γδ T cells have previously been described as “NK-like” γδ T cells (25). No difference between TB cases and LTBI contacts was seen in the proportion of CD3^lo^γδ cells, but LTBI subjects had a significantly higher proportion of CD3^bright^ γδ T cells compared to active TB patients (Figure 3B). Both CD3^bright^ and CD3^lo^ γδ T cells were able to produce IFN-γ after PMA/ionomycin stimulation, but IL-17 production both alone and in conjunction with IFN-γ was restricted to the CD3^lo^ subset (Figure 3C). No significant difference was observed between TB cases and LTBI subjects in the proportions of Vγ9δ2 T cells producing either IFN-γ or IL-17 alone; however, a significantly lower proportion of CD3^lo^γδTCR^+^IFN-γ^+^IL-17^+^ cells was seen in TB cases compared to LTBI (median [IQR] = 0.03% [0.00–0.21%] for active TB compared to 0.32% [0.05–1.18%] for LTBI; *p* = 0.0252; Figure 3D). No differences were seen between IGRA NC and C (data not shown).

### Production of IL-17 from Vα7.2^+^CD161^+^ Cells

**Figure 4:**
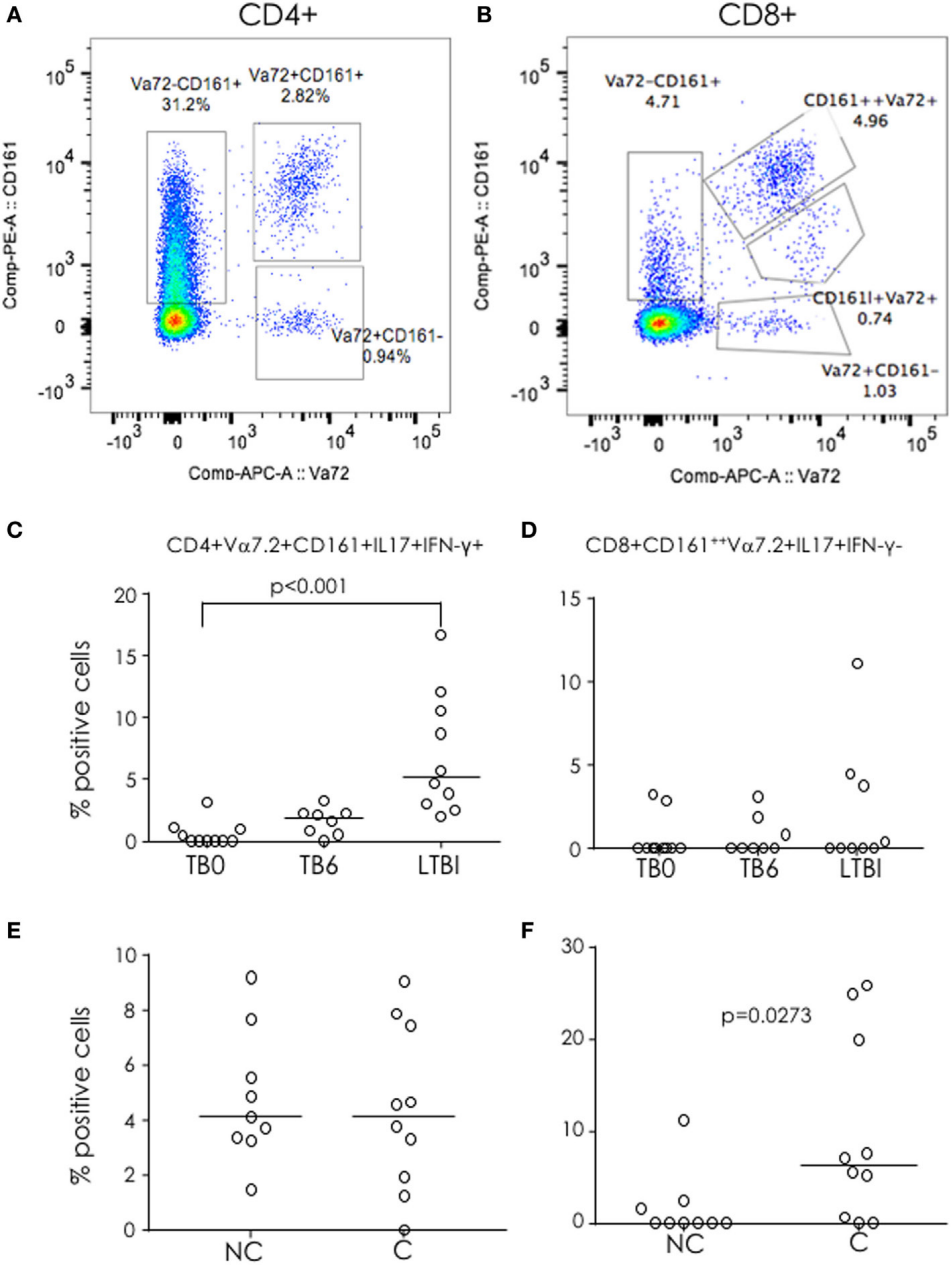
Functional Vα7.2^+^CD161^+^ cells in tuberculosis (TB) infection and disease. (A,B) Representative FACS plots for CD161 and Vα7.2 expression within CD4^+^ (**A**) and CD8^+^ (**B**) cells. Three defined CD4^+^ subsets and four defined CD8^+^ subsets (boxes) were analyzed for cytokine production (IL-17 and IFN-γ). Proportion of IL-17^+^IFN-γ^+^CD4^+^Vα7.2^+^CD161^+^ (**C,E**) and IL-17I^+^FN-g^+^ mucosal-associated invariant T cells (**D,F**) in TB cases before (TB0) and after treatment (TB6) and latently TB infected (**C,D**) and in IGRA non-converters (NC) and converters **(C)** (**E,F**). Data were analyzed using Kruskal–Wallis test (**C,D**) or Mann–Whitney *U*-test **(E,F)**. Bar indicates median.

Invariant CD8^+^ cells have previously been implicated in protective immunity to TB (12). Thus, we analyzed differential expression of Vα7.2 and CD161 within both CD4^+^ and CD8^+^ T cell subsets. Within the CD4^+^ subset, four distinct populations were evident: Vα7.2^−^CD161^−^, Vα7.2^−^CD161^+^, Vα7.2^+^CD161^+^, and Vα7.2^+^CD161^−^ (Figure 4A). Within the CD8^+^ subset, five distinct populations were evident: Vα7.2^−^CD161^−^, Vα7.2^−^CD161^+^, Vα7.2^+^CD161^++^ (MAIT), Vα7.2^+^CD161^+^, and Vα7.2^+^CD161^−^ (Figure 4B). We found no difference in the proportions of each of the phenotypic subsets between TB cases and LTBI or between IGRA C and NC (data not shown). However, when functionality was assessed, there was a significantly higher proportion of CD4^+^Vα7.2^+^CD161^+^ cells producing both IL-17 and IFN-γ in LTBI compared to pre-treatment (*p* < 0.001) active TB cases (Figure 4C) but no difference between IGRA C and NC (Figure 4E). In addition, no difference was seen in TB cases compared to LTBI (Figure 4D) but there was a significantly higher proportion of CD8^+^ MAIT cells producing IL-17 alone in IGRA C compared to NC (*p* = 0.0273; Figure 4F).

### Differential Mtb-Specific Production of Th17 Family Cytokines in IGRA C and NC

**Figure 5:**
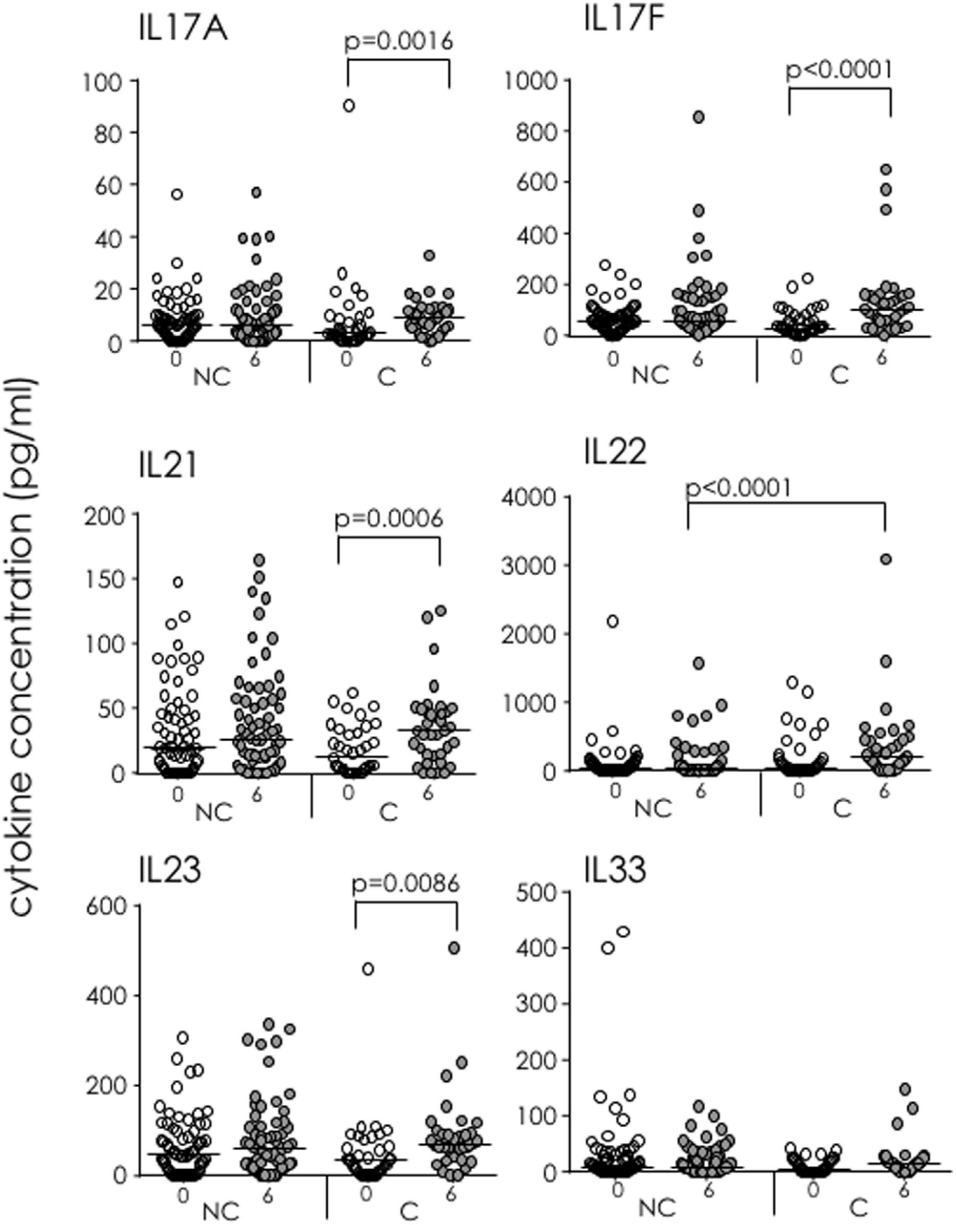
Soluble IL-17 superfamily factors in IFN-γ-release assay (IGRA) converters (C) and non-converters (NC) following purified protein derivative (PPD) stimulation of whole blood. 15-plex Th17 cytokine profiles were analyzed following PPD stimulation of whole blood from IGRA NC and C at recruitment (white dots) and 6 months (gray dots). Showing IL-17A, IL-17F, IL-21, IL-22, IL-23, and IL-33. Data were analyzed using Kruskal–Wallis test followed by Dunn’s post-test comparison. Bar indicates median.

Following overnight stimulation of whole blood with PPD, significantly higher concentrations of IL-17A, IL-17F, IL-21, and IL-23 were produced by recently infected IGRA C at 6 months compared to their preinfection (baseline) concentrations (*p* = 0.0016, *p* < 0.0001, *p* = 0.0006, and *p* = 0.0086, respectively) (Figure 5). No changes in PPD-induced cytokine concentrations were seen between baseline and 6 months for IGRA NC, and apart from significantly higher concentrations of IL-22 at 6 months in IGRA C compared to NC (*p* < 0.0001), there were no significant differences between the IGRA C and NC at either time point. There were no differences in IL-33 production either within or between groups (Figure 5).

**Figure 6:**
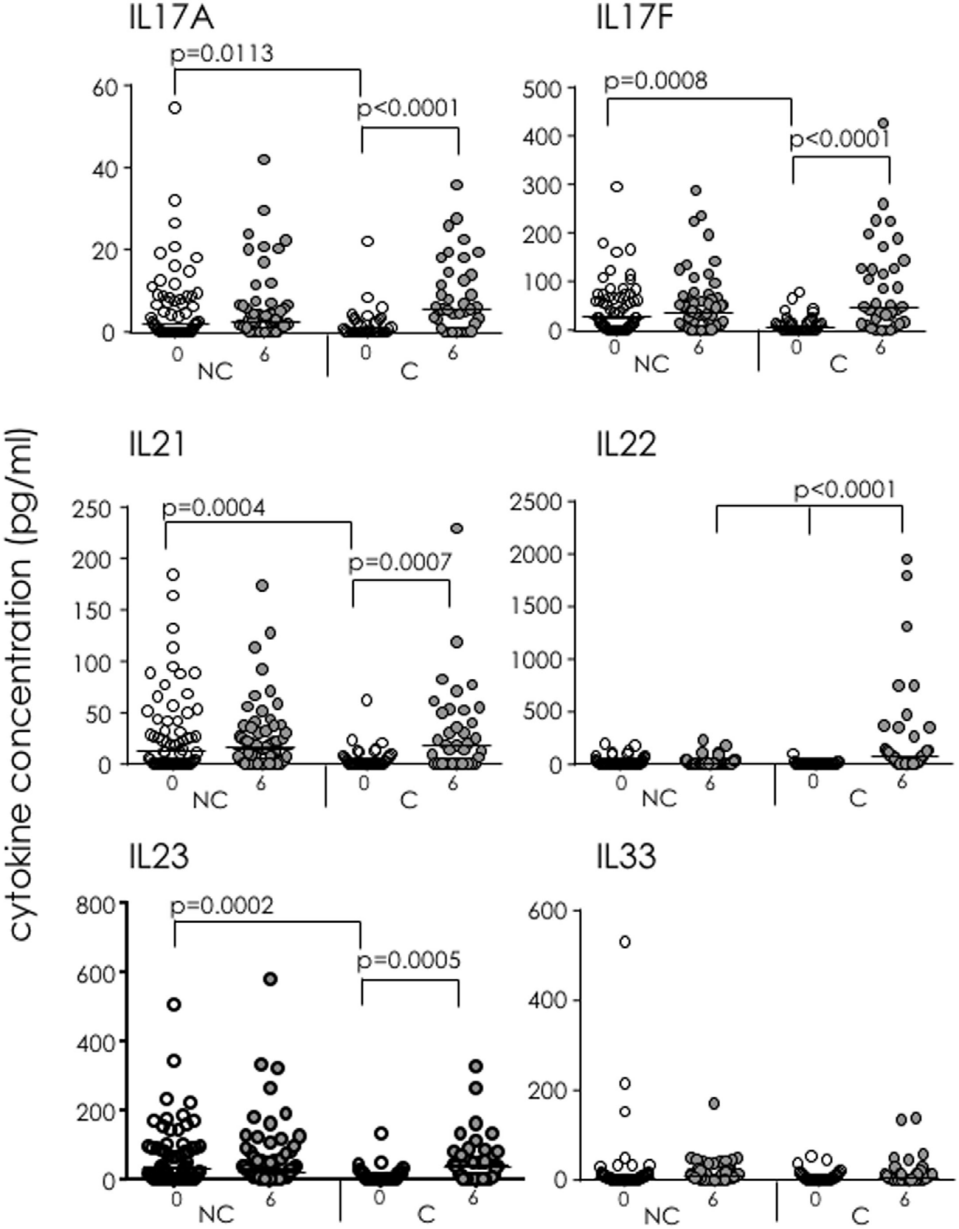
Soluble IL-17 superfamily factors in IFN-γ-release assay (IGRA) converters (C) and non-converters (NC) following ESAT-6/CFP-10 stimulation of whole blood. 15-plex Th17 cytokine profiles were analyzed following EC stimulation of whole blood from IGRA NC and C at recruitment (white dots) and 6 months (gray dots). Showing IL-17A, IL-17F, IL-21, IL-22, IL-23, and IL-33. Data were analyzed using Kruskal–Wallis test followed by Dunn’s post-test comparison. Bar indicates median.

Following overnight stimulation of whole blood with EC, concentrations of IL-17A, IL-17F, IL-21, and IL-23 were all significantly higher in IGRA NC than IGRA C at baseline (*p* = 0.0113, *p* = 0.0008, *p* = 0.0004, and *p* = 0.0002, respectively; Figure 6). Cytokine concentrations did not differ between baseline and 6 months for IGRA NC, but there were significant increases in concentrations of all four of these cytokines from baseline to 6 months among IGRA C. Moreover, EC-specific IL-22 production was observed only in IGRA C at 6 months, suggesting that EC-specific IL-22 is a biomarker of infection in some household contacts of TB cases. No differences in IL-33 concentrations were seen within or between groups.

## Discussion

The role of IL-17 and its family members in protective immunity to TB remains unclear. In addition, while the role of IL-17-producing cells, including Th17, MAIT, and γδ T cells has been extensively studied in mice, there are little data available in humans, particularly from West Africa. Therefore, in this study, we aimed to determine the role of soluble IL-17 and IL-17-producing cells at three key transition points in the Mtb infection spectrum: (i) after exposure to a TB case but prior to development of latent TB infection, (ii) after development of LTBI but prior to development of active TB disease, and (iii) before and after treatment of active TB disease. We found the main source of IL-17 was from CD26^+^ Th17 cells in all subjects. However, while secreted IL-17 levels were higher in TB-exposed IGRA NC, they had a lower proportion of IL-17^+^ cells compared to IGRA C at baseline (i.e., prior to conversion/infection). In active TB patients, cellular levels of IL-17 were significantly lower than in LTBI.

We found relatively high levels of IFN-γ^+^IL-17^+^ dual-producing CD4^+^ T cells across all participant groups. In a previous study of TB patients, accumulation of cells producing both Th1/Th17 cytokines correlated with disease severity (8). However, we saw significantly lower levels of IFN-γ^+^IL-17^+^ dual-producing invariant (Vα7.2^+^CD161^+^) CD4^+^ T cells and also IL-17^+^IFN-γ− Th17 cells in active TB compared to LTBI, suggesting that in our Gambian population, IL-17-producing CD4^+^ T cells either alone or in combination with IFN-γ have either migrated to the lung or are not pathogenic in our setting. Several studies have shown differential soluble and cellular cytokine production in active compared to LTBI (26–28). While levels are generally higher in active TB indicating a pathogenic effect [i.e., TNF-α (26), polyfunctional T cells (27)], in line with our findings, a study from Malawi showed higher soluble IL-17 in LTBI compared to active TB (28). One limitation of our study is that we used a polyclonal stimulant to induce IL-17 production, and thus, we may see differences with Mtb antigen-specific stimulation, currently under investigation in our laboratory.

In highly TB-exposed contacts, those that converted to a positive IGRA by 6 months had a significantly higher proportion of IL-17-producing CD4^+^CD26^−^ cells compared to NC at baseline. These cells have been found to be abundant in autoimmune diseases, cancers, and also in reactive tissues (29), suggesting that they are potential inflammatory response markers in Mtb infection. Interestingly, we saw significantly lower levels of both cell types in active TB pretreatment compared to LTBI, supporting previous findings that IL-17 is increased during early Mtb infection (i.e., prior to disease progression). We saw no difference in the total proportion of CD4^+^ or CD8^+^CD161^+^Vα7.2^+^ cells in IGRA NC and C. However, similar to Th17 cells, the proportions of MAIT cells producing IL-17 alone were also significantly lower in IGRA NC compared to IGRA C at baseline. This is possibly due to migration of cells from peripheral blood into the tissues but could not be determined in this study. Interestingly, we saw no CD4^−^CD8^−^ double negative MAIT cells in our setting (data not shown), but we did see a distinct population of CD4^+^Vα7.2^+^CD161^+^ cells, which could potentially be germline-encoded mycolyl-reactive cells (30). Our study supports previous findings on production of IL-17 by MAIT cells following PMA stimulation (31), which has not been seen in other African countries (Wong et al., manuscript in preparation). This may be due to different Mtb strains or host genetics.

In contrast to studies in mice (4), we found virtually no IL-17 production from γδ T cells. Previous reports have shown that γδ T cells represent several distinct subsets, which may differ in the stimuli needed to bring about their response (32). Certainly, the majority of findings in healthy donors have observed that the predominant subset of γδ T cells present in the human peripheral blood, characterized by the expression of a Vγ9Vδ2 TCR, contained very few (typically <1%) IL-17 producers (20). However, in bacterial meningitis, up to 60–70% of Vγ9Vδ2 T cells were IL-17^+^ when cultured using Th17 polarizing conditions (20). Another report has shown that IL-17-producing γδ T cells were significantly increased in TB patients compared to healthy donors (16), and studies in mice have shown that stimulation in IL-17-promoting conditions (i.e., exogenous IL-23) results in higher production of IL-17 from γδ T cells than from Th17 cells (32). Interestingly, no difference was seen in the proportion of “NK-like” γδ T cells (CD3^lo^) (25) between groups, but IL-17 production both alone and in conjunction with IFN-γ was restricted to this subset unlike previous reports in mice showing IL-17 production solely from CD3^bright^ γδ T cells (33).

In IGRA NC, levels of soluble IL-17A, IL-17F, IL-21, and IL-23 in EC-stimulated blood were significantly higher at baseline than in IGRA C, suggesting production precedes early clearance of the pathogen, most likely through innate cells. In contrast, IL-22 was only detectable in participants who had recently converted to a positive IGRA test results. IL-22 is an effector cytokine of IL-17 that acts directly on the lung epithelia (34) and has been shown to inhibit growth of Mtb in human macrophages (35). This supports a protective role of IL-17 and IL-22 during early infection and suggests IL-22 as a potential marker of LTBI. It is important to note that our data on soluble mediators used whole blood stimulation with antigen-specific stimulation, while our cellular analysis used PBMC with polyclonal stimulation, suggesting that the inclusion of neutrophils and other innate cells in the stimulation may further promote IL-17 production. In addition, it suggests that production of IL-17 is cytokine mediated (i.e., through promotion of IL-22 and IL-23) rather than TCR mediated.

In conclusion, these data provide new insights into the role of IL-17 and IL-17-producing cells in the pathogenesis of TB. Our findings suggest differential roles for IL-17-producing cells at distinct stages of the TB infection spectrum and can potentially be exploited for novel diagnostics and therapeutics. Future longitudinal studies will analyze responses following Mtb antigen-specific stimulation and using Th17-polarizing conditions and will attempt to determine whether Th17 cells play a role in protection from TB infection and/or disease.

## Ethics Statement

All subjects gave written informed consent in accordance with the Declaration of Helsinki. The protocol was approved by the MRCG/Gambian government joint ethics committee.

## Author Contributions

FC, AP, DM, JM, and MG performed experiments and analyzed data; JS developed the concept, designed the study, analyzed data, and wrote the paper; DL, BK, and ER provided conceptual advice and all authors commented on the paper.

## Conflict of Interest Statement

The authors declare that the research was conducted in the absence of any commercial or financial relationships that could be construed as a potential conflict of interest.
